# Clinical characteristics and serological profiles of Lyme disease in children: a 15-year retrospective cohort study in Switzerland

**DOI:** 10.1016/j.lanepe.2024.101143

**Published:** 2024-12-09

**Authors:** Beat M. Greiter, Semjon Sidorov, Ester Osuna, Michelle Seiler, Christa Relly, Annette Hackenberg, Isabelle Luchsinger, Elvira Cannizzaro, Roland Martin, Martina Marchesi, Stefanie von Felten, Adrian Egli, Christoph Berger, Patrick M. Meyer Sauteur

**Affiliations:** a Division of Infectious Diseases and Hospital Epidemiology, Children's Research Center, University Children's Hospital Zurich, University of Zurich, Zurich, Switzerland; b Emergency Department, University Children's Hospital Zurich, Zurich, Switzerland; c Department of Pediatric Neurology, University Children's Hospital Zurich, Zurich, Switzerland; d Department of Dermatology, Pediatric Skin Center, University Children's Hospital Zurich, Zurich, Switzerland; e Department of Rheumatology, University Children's Hospital Zurich, Zurich, Switzerland; f Institute of Experimental Immunology, University of Zurich, Zurich, Switzerland; g Institute of Medical Microbiology, University of Zurich, Zurich, Switzerland; h Medica-Medical Laboratories, Zurich, Switzerland; i Department of Biostatistics at Epidemiology, Biostatistics and Prevention Institute (EBPI), University of Zurich, Zurich, Switzerland

**Keywords:** Borrelia burgdorferi, Erythema migrans, Facial nerve palsy, Meningitis, Neuroborreliosis, Lyme arthritis

## Abstract

**Background:**

Lyme disease (LD) is caused by *Borrelia burgdorferi* and is the most common tickborne disease in the northern hemisphere. Although classical characteristics of LD are well-known, the diagnosis and treatment are often delayed. Laboratory diagnosis by serological testing is recommended for most LD manifestations. The objective of this study was to describe clinical characteristics and associated serological profiles in children with LD.

**Methods:**

This retrospective cohort study included children aged 0–18 years, diagnosed with LD according to current guidelines at University Children's Hospital Zurich between January 1, 2006 and December 31, 2020. Two-tier serological testing with the *recom*Well enzyme-linked immunosorbent assay and *recom*Line Western blot (MIKROGEN Diagnostik, MIKROGEN GmbH, Neuried, Germany) was performed at the Institute of Medical Microbiology, University of Zurich.

**Findings:**

In total, 469 children diagnosed with LD were included (median age, 7.9 years); 190 patients (40.5%) with Lyme neuroborreliosis (LNB), 171 (36.5%) patients with skin manifestations (erythema migrans, *n* = 121; multiple erythema migrans, *n* = 11; borrelial lymphocytoma, *n* = 37; and acrodermatitis chronica atrophicans, *n* = 2), and 108 (23.0%) patients with Lyme arthritis. We observed seasonal variations for patients with skin manifestations and LNB, with high prevalence in May–October, but not for patients with Lyme arthritis. Significant differences between LD manifestation groups were found for age, inflammatory parameters, and specificity and concentration of *B. burgdorferi*-specific serum antibody responses. We observed distinct patterns of pronounced serum antibody responses against *B. burgdorferi* antigens in LNB (IgM against VlsE, p41, and OspC) and Lyme arthritis (IgG against p100, VlsE, p58, p41, p39, and p18).

**Interpretation:**

Our study is one of the largest and most detailed for children with LD. We present unique findings regarding the differences in clinical characteristics and immune responses between various manifestations of LD in children.

**Funding:**

No specific funding to disclose for this study.

**Research in context**

**Evidence before this study**

We searched PubMed for articles published from January 1, 2006 to June 30, 2024. We used the MeSH terms “Lyme disease”, “*Borrelia burgdorferi*”, “borreliosis”, “erythema migrans”, “lymphocytoma”, “lymphadenosis benigna cutis”, “acrodermatitis chronica atrophicans”, “neuroborreliosis”, “facial nerve palsy”, “meningitis”, “radiculitis”, “Bannwarth's syndrome”, “arthritis”, or “carditis”. We prioritized original research articles or case series (at least five cases), but we also included systematic reviews. We found predominantly observational retrospective studies and case series that investigated the clinical presentation and serological responses in Lyme disease. However, most studies did not only include children, did focus on a single manifestation (e.g., Lyme neuroborreliosis), or the serological analyses were limited (e.g., two-tier serological testing with enzyme-linked immunosorbent assay and Western blot, testing of blood and cerebrospinal fluid with evaluation of intrathecal antibody production in patients with Lyme neuroborreliosis). Moreover, to our knowledge, there was no study comparing detailed serologic results between different manifestations in a large cohort of children with Lyme disease.

**Added value of this study**

This study describes clinical characteristics and serological profiles in a well-defined retrospective cohort of 469 children with Lyme disease in Switzerland. Two-tier serological testing for the detection of *B. burgdorferi-*specific antibodies in serum and cerebrospinal fluid was performed throughout the 15-year study period with the same well-established assays (*recom*Well enzyme-linked immunosorbent assay and *recom*Line Western blot, MIKROGEN Diagnostik, MIKROGEN GmbH, Neuried, Germany). To our knowledge, this study represents the largest clinical and serological data set for children with Lyme disease. Additionally, this work provides a deep dive into different Lyme disease manifestations and associated serological profiles.

**Implications of all the available evidence**

This study of Lyme disease in children presents unique findings regarding the differences in clinical characteristics and associated serological profiles between various manifestations. We found that distinct patterns of pronounced serum antibody responses of the IgM isotype against three *B. burgdorferi* antigens (VlsE, p41, and OspC) may help to predict Lyme neuroborreliosis, and of the IgG isotype against six *B. burgdorferi* antigens (p100, VlsE, p58, p41, p39, and p18) were associated with Lyme arthritis. Considering the diagnostic challenges in Lyme disease, and especially Lyme neuroborreliosis, there is a need to advance current testing methods while exploring new and innovative diagnostic approaches.

## Introduction

Lyme disease (LD) is the most common tickborne disease in the northern hemisphere.^1^ It is caused by several genospecies of the bacterium *B. burgdorferi* sensu lato complex (Lyme group *Borrelia* bacteria),^2^ nine of which are pathogenic for humans.^1^, ^2^, ^3^ In Europe, on average 15% of *Ixodes ricinus* ticks are infected with *B. burgdorferi*,^4^ and 3% of humans develop LD after a *I. ricinus* tick bite.^5^, ^6^, ^7^ LD cases occur mainly from May to October, reflecting peak tick feeding periods.^1^^,^^8^

The reported incidence of LD varies among regions and countries.^9^^,^^10^ In Europe, the main endemic areas are in Scandinavia and the southern part of central Europe. The annual incidence in these regions, considering all cases of LD among children and adults, is estimated to be 300 cases per 100,000 population.^1^ In Switzerland, 8000–15,000 new LD cases are estimated to occur per year.^11^ The highest rates of LD are reported in children aged 5–9 years and in adults aged 45–55 years.^8^^,^^12^ To our knowledge, the exact incidence of LD in children remains unclear.^13^

The clinical presentation of LD in children is variable.^14^ It most commonly affects the skin, nervous system, joints and, less commonly, the heart.^1^^,^^8^^,^^13^^,^^15^ Typical skin manifestations of LD in children in Europe include erythema migrans (EM; an expanding erythema that develops into a large erythema with a bright red outer border and central clearing) and borrelial lymphocytoma (painless bluish-red nodule).^13^ Acrodermatitis chronica atrophicans is an uncommon skin manifestation in children.^13^^,^^16^^,^^17^ Children with Lyme neuroborreliosis (LNB) primarily present with cranial nerve impairment and lymphocytic meningitis.^18^ Meningoradiculitis, also referred to as Bannwarth's syndrome or Garin-Bujadoux-Bannwarth syndrome,^19^ which is often seen in adults with LNB, has been rarely described in children.^13^^,^^16^ Data about children with Lyme arthritis in Europe are rare.^20^ Lyme arthritis occurs usually weeks to months after a tick bite, and mostly affects large joints.^21^ Heart manifestations are rare in children and most frequently present as carditis.^15^

The diagnosis of LD is based on signs and symptoms and the detection of *B. burgdorferi*-specific antibodies in serum, and for LNB, also in cerebrospinal fluid (CSF).^1^ The demonstration of intrathecal antibody production (IAP) against *B. burgdorferi* in CSF has been considered a gold standard for the diagnosis of LNB.^22^^,^^23^ Current guidelines recommend serological testing for all manifestations of LD, except for EM where antibodies against *B. burgdorferi* are rarely detectable at this early stage.^22^^,^^23^ However, identifying LD in children can be very challenging. The clinical presentations of LD can be similar to those of other pediatric diseases like juvenile idiopathic arthritis or idiopathic peripheral facial nerve palsy (Bell's palsy).^13^^,^^21^ Characteristic signs and symptoms can occur before *B. burgdorferi*-specific antibodies are detectable.^18^ Further, the major limitation of serological testing is that serum antibodies against *B. burgdorferi* remain for months to years after primary infection.^8^ Also, current diagnostic guidelines focusing on adults have not yet been validated for children.^13^^,^^22^^,^^23^ This is, at least in part, due to a lack of accurate data on clinical and immunological features of LD in children.

The objective of this study was to describe detailed epidemiological, clinical, and laboratory characteristics, and to explore the associated serological profiles of different manifestations of LD in children.

## Methods

### Cohort definition

**Fig. 1:**
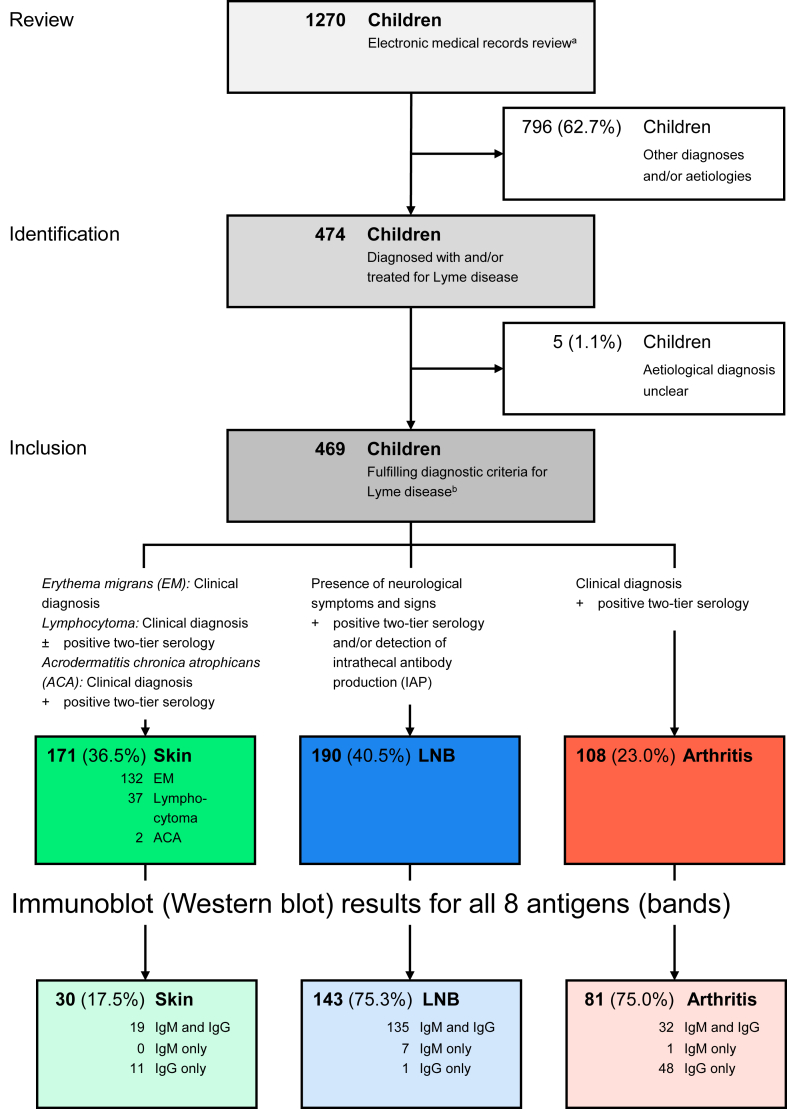
**Study flow.**^a)^ Search terms (in alphabetical order): “acrodermatitis chronica atrophicans”, “Bannwarth's syndrome”, “*Borrelia burgdorferi*”, “borreliosis”, “carditis”, “erythema migrans”, “erythemata migrantia”, “facial palsy”, “facial nerve palsy”, “Lyme”, “lymphadenosis benigna cutis”, “Lyme arthritis”, “Lyme borreliosis”, “Lyme disease”, “lymphocytoma”, “meningoradiculitis”, “neuroborreliosis”, and “radiculitis”. ^b)^ Inclusion criteria according to current guidelines for the diagnosis of LD^22^^,^^23^: (1) skin manifestations, including EM (clinical diagnosis); lymphocytoma (clinical diagnosis WITH/WITHOUT positive two-tier serology); and acrodermatitis chronica atrophicans (clinical diagnosis AND positive two-tier serology); (2) Lyme neuroborreliosis (LNB), i.e., LNB (presence of neurological symptoms and signs AND positive two-tier serology [serum and/or CSF] AND/OR IAP); and isolated peripheral facial nerve palsy (FP) (FP without any other medical symptoms and signs [e.g., headache, fever, nuchal rigidity, etc.]^18^); (3) Lyme arthritis (clinical diagnosis AND positive two-tier serology). All serological tests were performed at the Institute of Medical Microbiology, University of Zurich. Patients were categorized according to the organ system involved. Patients fulfilling the diagnostic criteria for LNB were considered to have LNB, regardless of whether skin manifestations such as EM were also observed. **Abbreviations:** EM, erythema migrans; IAP, intrathecal antibody production; LD, Lyme disease; LNB, Lyme neuroborreliosis.

This retrospective cohort study included children and adolescents ≤18 years of age diagnosed with LD at the University Children's Hospital Zurich between January 1, 2006 and December 31, 2020, which were identified through the electronic hospital database (Fig. 1).

First, the lists of diagnoses in electronic medical records of both inpatients and outpatients were screened by computer scientists applying the following search terms (in alphabetical order): “acrodermatitis chronica atrophicans”, “Bannwarth's syndrome”, “*B. burgdorferi*”, “borreliosis”, “carditis”, “erythema migrans”, “erythemata migrantia”, “facial palsy”, “facial nerve palsy”, “Lyme”, “lymphadenosis benigna cutis”, “Lyme arthritis”, “Lyme borreliosis”, “Lyme disease”, “lymphocytoma”, “meningoradiculitis”, “neuroborreliosis”, and “radiculitis”. Search results were also matched to ICD-10 diagnosis code A69.20 (“Lyme disease”) and to laboratory orders for *B. burgdorferi*-specific testing sent to the Institute of Medical Microbiology, University of Zurich (see below for details).

Next, the search results were reviewed by the study authors (B.M.G. and P.M.M.S.) and children with other diagnoses and/or aetiologies or where the aetiological diagnosis of LD was unclear were excluded.

Finally, only patients who fulfilled the diagnostic criteria according to current guidelines for the diagnosis of LD^22^^,^^23^ were included in the study: (1) skin manifestations, including EM (clinical diagnosis); lymphocytoma (clinical diagnosis WITH/WITHOUT positive two-tier serology); and acrodermatitis chronica atrophicans (clinical diagnosis AND positive two-tier serology); (2) Lyme neuroborreliosis (LNB), i.e., LNB (presence of neurological symptoms and signs AND positive two-tier serology [serum and/or CSF] AND/OR IAP); and isolated peripheral facial nerve palsy (FP) (FP without any other medical symptoms and signs [e.g., headache, fever, nuchal rigidity, etc.]^18^); (3) Lyme arthritis (clinical diagnosis AND positive two-tier serology).

Further, a subcohort was defined which included all patients with a positive serological test result consisting of a complete IgM and/or IgG Western blot for *all* 8 *B. burgdorferi* antigens.

Patients with LNB and isolated FP routinely underwent lumbar puncture during the 15-year study period. Follow-up visits over the 15-year study period were usually scheduled at the end of treatment for patients with LNB other than isolated FP, and at 10 days and 4 weeks after the onset of FP for patients with isolated FP. Otherwise, follow-up visits were individually planned and conducted differently in accordance with clinical manifestations and course.

### Clinical and laboratory data

Epidemiological, demographic, clinical, and laboratory data were extracted from medical records for each patient. Laboratory examination was performed as part of routine clinical care. The following parameters were considered: white blood cell count (WBC), C-reactive protein (CRP), serum glucose, CSF cell count, and CSF glucose, protein, and lactate.

After merging clinical and laboratory data, each patient was assigned a number. The coded data were transferred to a password-protected Microsoft Excel spreadsheet, ensuring controlled access, user rights, and change tracking. The key code will be destroyed after publication, and the data will be stored for 10 years.

### *B. burgdorferi*-specific testing

Two-tier serology for the detection of *B. burgdorferi-*specific antibodies (IgM and IgG) in serum and CSF was performed at the Institute of Medical Microbiology, University of Zurich, according to manufacturer's instructions using the same assays over the whole 15-year study period. For all serological samples, first-tier screening with an enzyme-linked immunosorbent assay (ELISA) was performed (*recom*Well *Borrelia* IgM and IgG, MIKROGEN Diagnostik, MIKROGEN GmbH, Neuried, Germany).

If the screening test was positive (IgM, IgG, or both IgM and IgG), the second-tier immunoblot (Western Blot; *recom*Line *Borrelia* IgM and IgG, MIKROGEN Diagnostik) for this isotype was performed for detection of specific antibodies to various *B. burgdorferi* antigens. Diagnostic bands for *B. burgdorferi* antigens were evaluated for color reaction and intensity after addition of 1:100 diluted patient serum. The number of diagnostic bands on the immunoblot increased over the 15-year study period to 8 *B. burgdorferi* antigens: p100, VlsE, p58, p41, p39, OspA, OspC, and p18. The intensity was graded using a Dynablot plus instrument with *recom*Scan software (MIKROGEN Diagnostik) at the ISO-certified Institute of Medical Microbiology as follows: 0 (no reaction, equal to the intensity of the cutoff for the control), 1+, 2+, 3+, and 4+. Intensities of at least 1+ were considered positive. When positive, each band received a certain point value (IgG: p100, 5 points; VlsE, 5 points; p58, 4 points; p41, 1 point; p39, 5 points; OspA, 5 points; OspC, 5 points; and p18, 5 points; for IgM: same point values, except for p39, 4 points; and OspC, 8 points). The immunoblot was considered positive if the sum value of points was >7. Point values and point values threshold were provided by the manufacturer.^24^ Final interpretation of the serologic assay was based on immunoblot results for both IgM and IgG isotypes, as interpreted by the Institute of Medical Microbiology.

For IAP determination, an immunoblot (*recom*Line *Borrelia* IgG, MIKROGEN Diagnostik) was performed on serum and CSF, and IAP was determined by comparing band patterns and intensity between the two, according to the manufacturer's instructions.^25^ Patients with band intensity index (CSF immunoblot band intensity/serum immunoblot band intensity) >1.5,^26^ or with new bands in CSF compared to serum, were considered IAP positive.

### Outcomes

We considered the manifestation of LD as an intermediate outcome, with some of the other characteristics as potential causes (tick bite and patient characteristics) and some of the others as potential effects (serological results, treatment, and clinical outcomes). Due to the retrospective nature of our study, much of the data in our cohort was not recorded in a standardized way, particularly clinical outcomes. As a result, we have not defined outcome and exposure variables. This study is therefore largely descriptive.

### Statistical analysis

Descriptive statistics of the whole cohort and of the subcohort (patients with a positive serological test result consisting of a complete IgM and/or IgG Western blot for *all* 8 *B. burgdorferi* antigens) were tabulated by LD manifestation (skin, LNB, arthritis). Median and interquartile range (first and third quartile) was reported for continuous variables and frequency and percentage for categorical variables.

To assess differences between the three groups, we performed Kruskal–Wallis rank sum tests for continuous variables and Fisher's exact tests for categorical variables and report unadjusted P-values. It is important to note that these P-values should be interpreted in an exploratory manner. Also, due to the presence of confounding, we do not claim that associations between clinical manifestation of LD and other characteristics are causal.

The mean incidence (2006–2020) and incidence rate (2006–2010 vs. 2016–2020) per 100,000 population was calculated for each time period based on the annual number of LD cases (numerator) and the annual population of the child and adolescent population in the Canton of Zurich (denominator). The term “specificity” in this study refers to the specificity of an antibody (and not the specificity of a test), defined by the ability of the antibody to recognize and bind its intended epitope (*B. burgdorferi* antigens).

Analyses were performed with R software, version 4.4.0 (R Foundation for Statistical Computing).

### Ethical considerations

This study was approved by the ethics committee of the Canton of Zurich (BASEC-Nr. 2022-02226). The general consent at the University Children's Hospital Zurich was introduced in 2014. Thus, this study was approved by the ethics committee as a further use of biological material and health-related personal data for research without consent.

## Results

### Study population

A total of 469 patients fulfilled the inclusion criteria of LD and were included into this study (Fig. 1). Patients were grouped according to the different manifestation of LD into (1) skin manifestations (*n* = 171); (2) LNB (*n* = 190); and (3) Lyme arthritis (*n* = 108) (Fig. 1 and Table 1). No cases of Lyme carditis were identified. The subcohort of patients with a positive serological test result consisting of a complete IgM and/or IgG Western blot for all 8 *B. burgdorferi* antigens included 254 patients (skin manifestations, *n* = 30; LNB, *n* = 143; and Lyme arthritis, *n* = 81).

**Table 1:** Clinical manifestations of Lyme disease among 469 Swiss children and adolescents aged ≤18 years grouped by skin manifestation, Lyme neuroborreliosis (LNB), and Lyme arthritis.

Manifestation	Diagnosis	Disease stage^a^	*n* (%)^b^
**Skin manifestation**	Total		171 (36.5)
	Erythema migrans (EM)	I	121 (25.8)
	Head/neck		38
	Trunk		37
	Upper limb		12
	Lower limb		31
	Unknown localization		3
	Multiple EM	II	11 (2.3)
	Lymphocytoma	II	37 (7.9)
	Ear lobe		27
	Supraorbital		3
	Mamilla		4
	Cheek		2
	Unknown location		1
	Acrodermatitis chronica atrophicans	III	2 (0.4)
**Lyme neuroborreliosis (LNB)**	Total		190 (40.5)
	Isolated peripheral facial nerve palsy (FP)^c^	II	63 (13.4)
	Meningitis^d^	II	103 (22.0)
	+ FP		69
	+ Cranial neuropathy other than FP^e^		5
	Cranial neuropathy other than FP^f^	II	4 (0.9)
	(Meningo-) Radiculitis^g^	II	15 (3.2)
	Myelitis^h^	II–III	3 (0.6)
	Cerebral vasculitis^i^	II	2 (0.4)
**Lyme arthritis**	Total		108 (23.0)
	Monoarticular	II–III	95 (20.3)
	Knee		83
	Ankle		2
	Hip		4
	Elbow		3
	Other		3
	Oligoarticular	II–III	13 (2.8)

Patients were categorized according to the organ system involved. Patients fulfilling the diagnostic criteria for LNB were considered to have LNB, regardless of whether skin manifestations such as EM were also observed.

**Abbreviations:** EM, erythema migrans; LNB, Lyme neuroborreliosis; FP, peripheral facial nerve palsy.

a Disease stages I–III: I, early localized disease; II, early disseminated disease; III, late disease.^1^

b Percentages are related to all 469 cases.

c Defined as FP without any other medical symptoms and signs (e.g., headache, fever, nuchal rigidity etc.).^18^

d Lymphocytic meningitis denotes a clinical syndrome with headache, fever, nausea, vomiting, neck/back stiffness, photophobia, phonophobia, and predominance of lymphocytes in cerebrospinal fluid.^1^^,^^8^

e Abducens nerve palsy (*n* = 4) and trochlearis nerve palsy (*n* = 1), all in combination with meningitis.

f Abducens nerve palsy (*n* = 2) and polyneuritis (*n* = 2) without meningitis.

g Meningoradiculitis, originally described as Bannwarth's syndrome,^19^ denotes a combination of CSF lymphocytic pleocytosis and painful radiculitis.^1^^,^^8^

h Myelitis manifests with paraparesis, sensitive and proprioceptive disorders, CSF lymphocytic pleocytosis, and parenchymal lesions of the spinal cord in MRI scan.^1^^,^^8^

i Cerebral (occlusive) vasculitis, defined as inflammation of the blood vessel wall of a cerebral artery, which primarily causes headache, encephalopathy, and stroke-like symptoms.^1^^,^^8^

### Epidemiological characteristics

**Fig. 2:**
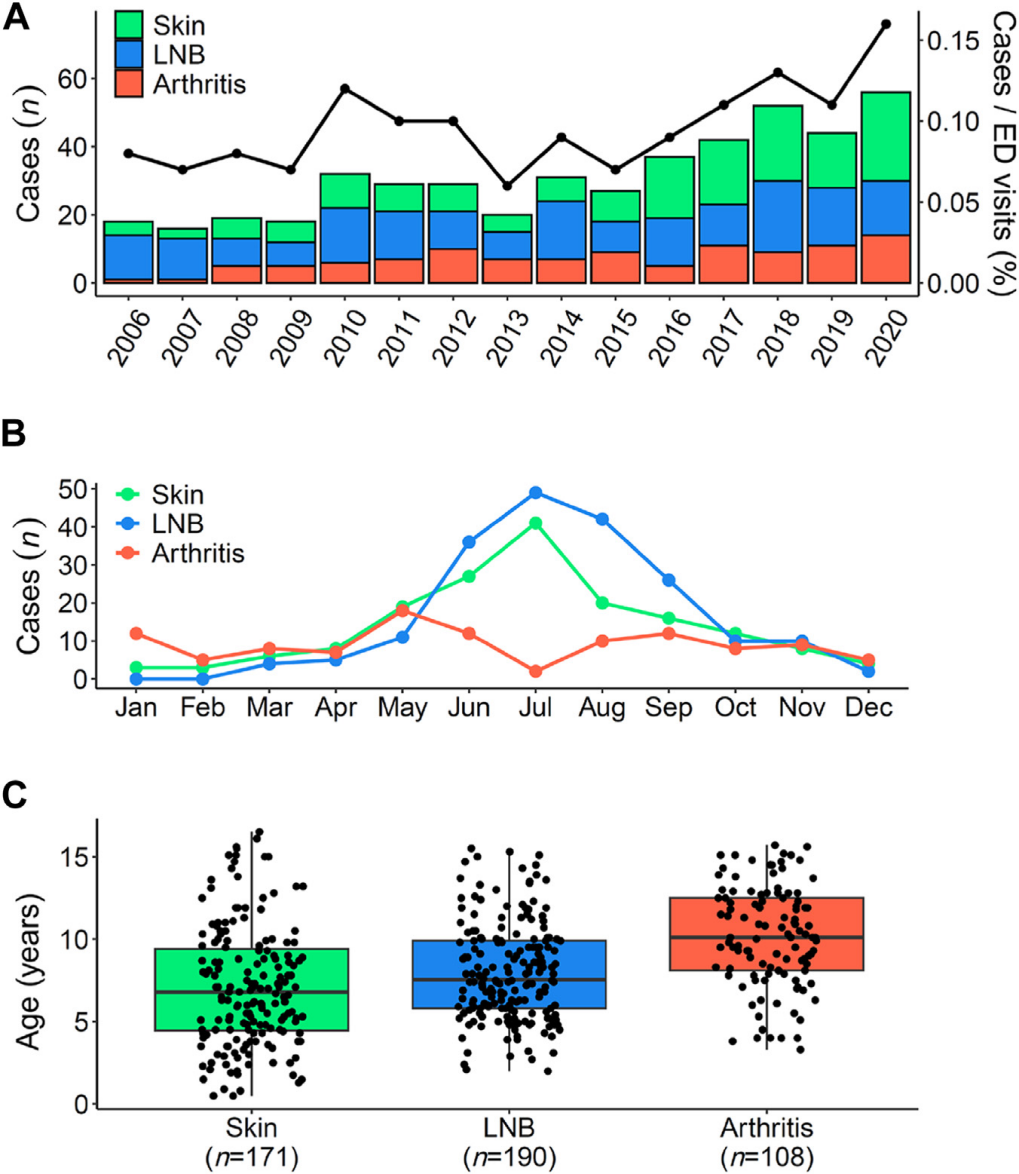
**Epidemiology and age distribution according to different manifestations of Lyme disease. A:** Cases per year. The black line illustrates annual cases normalized to total emergency department visits (%). **B:** Seasonal changes per month. Total number of cases over the 15-year study period per month. **C:** Age differences. The median is shown as a black line across the box that represents the lower and upper quartiles. Whiskers extend to the maximum and minimum values within 1.5 times the interquartile range above and below the third and first quartiles, respectively. **Abbreviations:** ED, emergency department; LNB, Lyme neuroborreliosis.

The annual number of included patients varied between 16 patients in 2007 and 56 patients in 2020 (Fig. 2A). The number increased from an average of 20 patients in the first 5 years (2006–2010) to 46 patients in the last 5 years of the study period (2016–2020; P = 0.04). The increase in patients was also corroborated in relation to the number of emergency department (ED) visits during the corresponding study period (Fig. 2A; black line). The mean incidence over the whole 15-year study period was 10.9 per 100,000 child and adolescent population in the Canton of Zurich,^27^ with an increase in incidence from 7.8 during 2006–2010 to 15.5 during 2016–2020.

LD was most frequently diagnosed from May to October, with peaks in June and July for skin manifestations and July and August for LNB (Fig. 2B). Lyme arthritis was diagnosed during the whole year with less fluctuation.

### Clinical characteristics

**Fig. 3:**
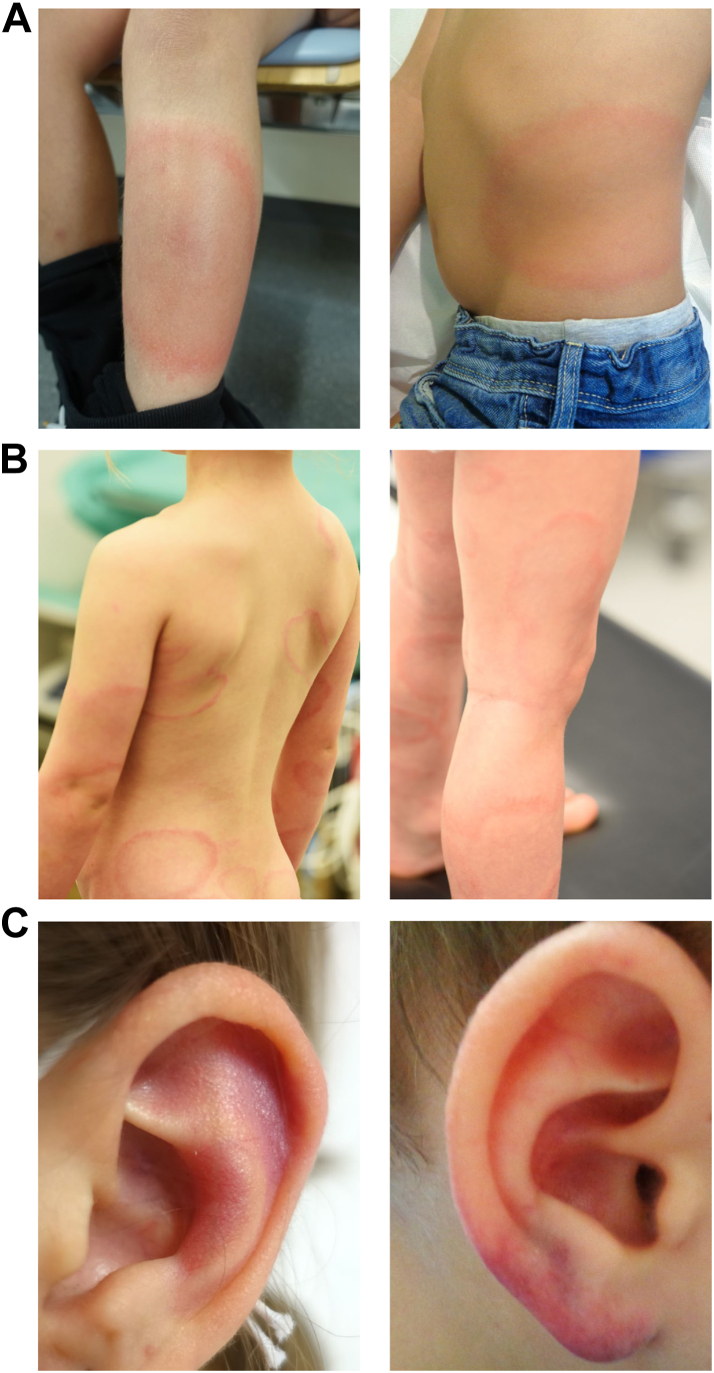
**Representative pictures of patients with erythema migrans and lymphocytoma. A:** Erythema migrans (EM). **B:** Multiple EM. **C:** Lymphocytoma.

Clinical manifestations are described in Table 1. EM was the most frequent presentation in patients with skin manifestations (*n* = 132/171, 77.2%; including multiple EM). Representative pictures of patients clinically diagnosed with EM and lymphocytoma are shown in Fig. 3. Among patients with LNB, most frequent presentations included meningitis (*n* = 103/190, 54.2%) and isolated FP (*n* = 63/190, 33.2%). Using the House–Brackmann classification of FP dysfunction as a clinical indicator of severity (grade I–V with increasing severity),^28^ the median grade was IV (IQR, III–V) (Supplementary Table S1). Lyme arthritis most frequently affected the knee (*n* = 83/108, 76.9%).

Detailed patient characteristics are shown in Table 2 and Supplementary Figure S1. The median age was 7.9 (IQR, 5.5–10.5) years and 266 (56.7%) patients were male. Clinical manifestations differed significantly regarding age, with Lyme arthritis patients being older than patients with skin manifestations and LNB (Table 2 and Fig. 2C). Patients <5 years (*n* = 87/469, 18.6%) predominantly presented with EM and lymphocytoma (*n* = 53/87, 60.9%). Underlying diseases were present in 52 (11.1%) patients. A tick bite was recognized in 203 (43.3%) of LD patients, and 164 (35.0%) reported a history of a rash that could be EM. The median time from recognized tick bite to ED presentation was 16.5 days (IQR, 11.0–40.0) for patients with skin manifestation (EM and lymphocytoma, *n* = 82), 30.0 days (IQR, 27.0–60.0) for LNB (*n* = 77), and 360 days (IQR, 180.0–720.0) for Lyme arthritis (*n* = 24) (P < 0.0001). We did not observe significant differences in time from symptom onset to ED presentation among manifestation groups (Table 2).

**Table 2:** Demographic characteristics and clinical presentation of 469 Swiss children and adolescents aged ≤18 years with Lyme disease grouped by skin manifestation, Lyme neuroborreliosis (LNB), and Lyme arthritis.

Characteristics	Total (*n* = 469)	Skin (*n* = 171)	LNB (*n* = 190)	Arthritis (*n* = 108)	P-value
**Demographic characteristics**					
Age (years)	7.9 (5.5, 10.5)	6.8 (4.4, 9.5)	7.6 (5.8, 9.9)	10.1 (8.1, 12.5)	<0.0001
Sex, male	266 (56.7%)	90 (52.6%)	113 (59.5%)	63 (58.3%)	0.41
Underlying disease	52 (11.1%)	19 (11.1%)	21 (11.1%)	12 (11.1%)	1.00
Underlying disease categories					0.95
Primary or secondary immunodeficiency	0 (0%)	0 (0%)	0 (0%)	0 (0%)	
Neurological	14 (26.9%)	4 (21.1%)	8 (38.1%)	2 (16.7%)	
Cardiovascular	5 (9.6%)	3 (15.8%)	1 (4.8%)	1 (8.3%)	
Pulmonary	6 (11.5%)	2 (10.5%)	3 (14.3%)	1 (8.3%)	
Gastrointestinal	3 (5.8%)	1 (5.3%)	1 (4.8%)	1 (8.3%)	
Others	24 (46.2%)	9 (47.4%)	8 (38.1%)	7 (58.3%)	
No underlying disease	417 (88.9%)	152 (88.9%)	169 (88.9%)	96 (88.9%)	
Exposition to tick bite					0.06
Farm	3 (2.9%)	2 (3.8%)	0 (0.0%)	1 (12.5%)	
Forest	65 (63.1%)	36 (69.2%)	26 (60.5%)	3 (37.5%)	
Garden	5 (4.9%)	2 (3.8%)	3 (7.0%)	0 (0.0%)	
Hiking	3 (2.9%)	0 (0.0%)	3 (7.0%)	0 (0.0%)	
Scout	3 (2.9%)	0 (0.0%)	3 (7.0%)	0 (0.0%)	
Unknown exposition	24 (23.3%)	12 (23.1%)	8 (18.6%)	4 (50.0%)	
NA	366	119	147	100	
Tick bite recognized	203 (43.3%)	91 (53.2%)	82 (43.2%)	30 (27.8%)	0.00014
Location of recognized tick bite					0.0005^a^
Head/neck	52 (25.6%)	29 (31.9%)	22 (26.8%)	1 (3.3%)	
Trunk	41 (20.2%)	24 (26.4%)	16 (19.5%)	1 (3.3%)	
Upper limb	4 (2.0%)	3 (3.3%)	1 (1.2%)	0 (0.0%)	
Lower limb	13 (6.4%)	11 (12.1%)	2 (2.4%)	0 (0.0%)	
Multiple tick bites	7 (3.4%)	3 (3.3%)	2 (2.4%)	2 (6.7%)	
Unknown location	86 (42.4%)	21 (23.1%)	39 (47.6%)	26 (86.7%)	
NA	266	80	108	78	
EM recognized	164 (35.0%)	130 (76.0%)	33 (17.4%)	1 (0.9%)	<0.0001
Location of recognized EM					<0.0001
Head/neck	55 (33.5%)	38 (29.2%)	17 (51.5%)	0 (0.0%)	
Trunk	47 (28.7%)	40 (30.8%)	7 (21.2%)	0 (0.0%)	
Upper limb	11 (6.7%)	11 (8.5%)	0 (0.0%)	0 (0.0%)	
Lower limb	33 (20.1%)	32 (24.6%)	1 (3.0%)	0 (0.0%)	
Multiple EM	12 (7.3%)	7 (5.4%)	5 (15.2%)	0 (0.0%)	
Unknown location	6 (3.7%)	2 (1.5%)	3 (9.1%)	1 (100.0%)	
NA	305	41	157	107	
**Clinical presentation**					
Time from recognized tick bite to ED presentation (days)	30.0 (15.0, 90.0)	16.5 (11.0, 40.0)	30.0 (27.0, 60.0)	360.0 (180.0, 720.0)	<0.0001
NA	286	89	113	84	
Time from symptom onset to ED presentation (days)	5.0 (2.0, 15.0)	4.0 (1.0, 21.0)	7.0 (3.0, 14.0)	4.0 (2.0, 14.0)	0.17
NA	2	1	0	1	
Fever	68 (14.5%)	13 (7.6%)	39 (20.6%)	16 (14.8%)	0.0017
Headache	119 (25.4%)	7 (4.1%)	109 (57.4%)	3 (2.8%)	<0.0001
Nausea/vomiting	37 (7.9%)	7 (4.1%)	30 (15.8%)	0 (0.0%)	<0.0001
Nuchal rigidity	37 (7.9%)	1 (0.6%)	36 (19.1%)	0 (0.0%)	<0.0001
Scalp touch sensitive	12 (2.6%)	2 (1.2%)	10 (5.3%)	0 (0.0%)	0.0089
Fatigue	68 (14.5%)	8 (4.7%)	60 (31.7%)	0 (0.0%)	<0.0001
Change in behaviour/character	34 (7.2%)	3 (1.8%)	31 (16.3%)	0 (0.0%)	<0.0001
EM at ED presentation	157 (33.5%)	131 (76.6%)	26 (13.7%)	0 (0.0%)	<0.0001
Location of EM at ED presentation					0.0035
Head/neck	55 (35.0%)	39 (29.8%)^b^	16 (61.5%)^b^		
Trunk	40 (25.5%)	37 (28.2%)	3 (11.5%)		
Upper limb	13 (8.3%)	12 (9.2%)	1 (3.8%)		
Lower limb	32 (20.4%)	31 (23.7%)	1 (3.8%)		
Multiple EM	16 (10.2%)	11 (8.4%)	5 (19.2%)		
Unknown location	1 (0.6%)	1 (0.8%)	0 (0.0%)		
NA	312	40	164	108	
Lymphocytoma at ED presentation	37 (7.9%)	36 (21.1%)	1 (0.5%)	0 (0.0%)	<0.0001
Location of lymphocytoma at ED presentation					1.00
Ear lobe	27 (73.0%)	26 (72.2%)	1 (100.0%)		
Supraorbital	3 (8.1%)	3 (8.3%)	0 (0.0%)		
Mamilla	4 (10.8%)	4 (11.1%)	0 (0.0%)		
Cheek	2 (5.4%)	2 (5.6%)	0 (0.0%)		
Unknown location	1 (2.7%)	1 (2.8%)	0 (0.0%)		
NA	432	135	189	108	

Continuous variables are summarized as median (1st quartile, 3rd quartile), categorical variables as no. (%). P-values were calculated by the Kruskal–Wallis rank sum test (continuous variables) or Fisher's exact test (categorical variables).

**Abbreviations:** ED, emergency department; EM, erythema migrans; LNB, Lyme neuroborreliosis; NA, not available.

a P-value from Fisher's exact test calculated using Monte Carlo simulation.

b Including erythema on the cheeks (which resembles slapped-cheek rash from fifth disease due to parvovirus B19 infection).

Antibiotic regimen and treatment duration for each LD manifestation according to international guidelines at the respective time^9^ are shown in Table 3. Time from symptom onset to start of antibiotic treatment was a median of 4.0 days (IQR, 1.0–21.0) for patients with skin manifestations. Patients with LNB and Lyme arthritis had a longer delay in treatment initiation with a median of 19.0 days (IQR, 13.5–30.0) and 14.0 days (IQR, 9.0–27.0), respectively. Side effects were reported with ceftriaxone (*n* = 17, 3.8%), amoxicillin (*n* = 2, 0.5%), and amoxicillin with clavulanic acid (*n* = 2, 0.5%), with exanthema being the most common side effect for all antibiotic agents. Corticosteroids (prednisolone, 1–2 mg/kg, daily for 7 days) were given to 87.1% (*n* = 115/132) patients presenting with FP (isolated or in combination with other symptoms) within 5 days after symptom onset based on the evidence of benefit from corticosteroids in adults with Bell's palsy,^29^ although this has recently been shown to be less true in children.^30^

**Table 3:** Treatment and clinical outcome of 469 Swiss children and adolescents aged ≤18 years with Lyme disease grouped by skin manifestation, Lyme neuroborreliosis (LNB), and Lyme arthritis.

Characteristics	Total (*n* = 469)	Skin (*n* = 171)	LNB (*n* = 190)	Arthritis (*n* = 108)	P-value
**Treatment**					
Prior antibiotic treatment for LD	35 (7.5%)	14 (8.2%)	17 (8.9%)	4 (3.7%)	0.22
Prior antibiotic treatment duration (days)	3.5 (1.0, 6.0)	2.0 (1.0, 5.5)	4.0 (1.0, 6.0)	5.0 (4.0, 20.0)	0.24
NA	439	159	175	105	
Time from symptom onset to start of antibiotic treatment after ED presentation (days)	14.0 (5.0, 27.0)	4.0 (1.0, 21.0)	19.0 (13.5, 30.0)	14.0 (9.0, 27.0)	<0.0001
NA	37	8	26	3	
Antibiotic treatment after ED presentation					
Amoxicillin PO	138 (31.2%)	123 (73.7%)	1 (0.6%)	14 (13.0%)	
Ceftriaxone IV^a^	144 (32.6%)	0 (0.0%)	132 (79.0%)	12 (11.1%)	
Doxycycline PO	149 (33.7%)	35 (21.0%)	33 (19.8%)	81 (75.0%)	
Other antibiotic treatment^b^	11 (2.5%)	9 (5.4%)	1 (0.6%)	1 (0.9%)	
NA	27	4	23	0	
Antibiotic treatment duration (days)	21.0 (14.0, 21.0)	14.0 (14.0, 14.0)	21.0 (14.0, 21.0)	28.0 (28.0, 28.0)	
NA	32	4	28	0	
Side effects^c^					
Amoxicillin	2 (0.5%)	2 (1.2%)	0 (0.0%)		
Ceftriaxone	17 (3.8%)	0 (0.0%)	17 (10.2%)		
Amoxicillin with clavulanic acid	2 (0.5%)	2 (1.2%)	0 (0.0%)		
Doxycycline	0 (0%)	0 (0%)	0 (0%)		
No side effects	421 (95.2%)	163 (97.6%)	150 (89.8%)	108 (100.0%)	
NA	27	4	23	0	
Change of antibiotics					
To doxycycline	4 (57.1%)	1 (50.0%)	3 (60.0%)		
To penicillin	2 (28.6%)	0 (0.0%)	2 (40.0%)		
To clarithromycin	1 (14.3%)	1 (50.0%)	0 (0.0%)		
NA	462	169	185	108	
Prior corticosteroid treatment	12 (2.6%)	1 (0.6%)	11 (5.8%)	0 (0.0%)	0.001
Corticosteroids at presentation	115 (25.1%)		115 (63.2%)^d^		
**Clinical outcome**					
Hospitalization	43 (9.2%)	0 (0%)	13 (6.8%)	30 (27.8%)	<0.0001
Duration of hospital stay (days)	5.5 (4.0, 7.0)		6.5 (3.5, 9.0)	5.0 (4.0, 6.0)	0.26
NA	427^e^	171	178	78	
Total duration of symptoms (days)	40.0 (23.0, 66.0)	43.0 (22.0, 79.0)^f^	33.0 (23.0, 54.0)	45.0 (24.0, 114.0)	0.025
NA	263	148	93	22	
Presence of prolonged symptoms					<0.0001
2–3 months	4 (1.1%)	3 (2.2%)		1 (1.0%)	
4–6 months	9 (2.5%)	3 (2.2%)		6 (6.3%)	
>6 months	13 (3.6%)	1 (0.7%)		12 (12.5%)	
No prolonged symptoms	333 (92.8%)	128 (94.8%)	128 (100.0%)	77 (80.2%)	
NA	110	36	62	12	
Clinical outcome at last follow-up visit					0.63
Full recovery	359 (99.7%)	135 (100.0%)	128 (99.2%)	96 (100.0%)	
Abnormal clinical outcome^g^	1 (0.3%)	0	1 (0.8%)	0	
NA	109	36	61	12	

Continuous variables are summarized as median (1st quartile, 3rd quartile), categorical variables as no. (%). P-values were calculated by the Kruskal–Wallis rank sum test (continuous variables) or Fisher's exact test (categorical variables).

**Abbreviations:** LD, Lyme disease; IV, intravenous; LNB, Lyme neuroborreliosis; NA, not available; PO, peroral.

a Ceftriaxone IV once daily was administered on an outpatient basis at our day clinic for the entire duration of the study, unless the patient's general condition was severely compromised and required hospitalization with monitoring.

b Antibiotic treatment with other agents: skin: amoxicillin with clavulanic acid (*n* = 7), clarithromycin (*n* = 1), penicillin (*n* = 1); LNB: amoxicillin with clavulanic acid (*n* = 1); arthritis: flucloxacillin (*n* = 1).

c Amoxicillin: exanthema (*n* = 2); ceftriaxone: exanthema (*n* = 12), angioedema (*n* = 2), fever (*n* = 2), nausea and vomiting (*n* = 1), neutropenia (*n* = 5), elevated transaminases (*n* = 1); amoxicillin with clavulanic acid: exanthema (n = 1), neutropenia (*n* = 1).

d According to treatment recommendations indicated for and administered to the following patients: cranial neuropathies (if ≤5 days of symptoms), *n* = 113; (meningo-)radiculitis, *n* = 1; and myelitis, *n* = 1.

e The duration of hospital stay was unknown for one hospitalized patient.

f EM (*n* = 10), 22 days (7–34); lymphocytoma (*n* = 12), 76 days (47–103); acrodermatitis chronica atrophicans (*n* = 1), 437 days.

g Abnormal clinical outcome: cerebral vasculitis with cerebrovascular insult (*n* = 1).

The overall duration of symptoms varied significantly between manifestations: median 43 days for skin manifestation (IQR, 22.0–79.0), 33 days for LNB (23.0–54.0), 45 days for Lyme arthritis (24.0–114.0), (Table 3). Prolonged symptoms >6 months after initiation of antibiotic treatment were reported in 13 (3.6%) LD patients, 12 with Lyme arthritis and one with skin manifestation (acrodermatitis chronica atrophicans).

A full recovery was documented in 359 (99.7%) patients; one (0.3%) LNB patient with cerebral vasculitis and cerebrovascular insult experienced sequelae.

### Laboratory findings

Patients with Lyme arthritis showed significantly higher WBC counts, including neutrophils and monocytes, and CRP levels compared to patients with skin manifestations and LNB (Table 4 and Supplementary Figure S2).

**Table 4:** Laboratory findings at presentation of 469 Swiss children and adolescents aged ≤18 years with Lyme disease grouped by skin manifestation, Lyme neuroborreliosis (LNB), and Lyme arthritis.

Characteristics	Total (*n* = 469)	Skin (*n* = 171)	LNB (*n* = 190)	Arthritis (*n* = 108)	P-value
**Blood cell count**					
WBC count, G/L	8.0 (6.8, 9.4)	7.0 (5.5, 8.4)	7.6 (6.7, 9.3)	8.8 (7.3, 9.9)	0.00059
NA	189	148	17	24	
WBC count, categories^a^					0.27
Leukocytosis	13 (4.6%)	1 (4.3%)	5 (2.9%)	7 (8.3%)	
Leukopenia	7 (2.5%)	0 (0%)	6 (3.5%)	1 (1.2%)	
Normal	260 (92.9%)	22 (95.7%)	162 (93.6%)	76 (90.5%)	
NA	189	148	17	24	
ANC, G/L	4.3 (3.2, 5.7)	3.2 (2.4, 4.6)	4.0 (3.0, 5.4)	5.2 (4.2, 6.1)	<0.0001
NA	210	149	25	36	
ANC, categories^a^					0.52
Neutrophilia	0 (0%)	0 (0%)	0 (0%)	0 (0%)	
Neutropenia	8 (3.2%)	1 (4.5%)	6 (3.7%)	1 (1.5%)	
Normal	242 (96.8%)	21 (95.5%)	156 (96.3%)	65 (98.5%)	
NA	219	149	28	42	
Lymphocyte count, G/L	2.6 (1.9, 3.2)	2.9 (1.8, 3.5)	2.6 (2.0, 3.2)	2.3 (1.7, 3.0)	0.06
NA	210	149	25	36	
Lymphocyte count, categories^a^					0.41
Lymphocytosis	0 (0%)	0 (0%)	0 (0%)	0 (0%)	
Lymphopenia	12 (4.6%)	1 (4.5%)	6 (3.6%)	5 (6.9%)	
Normal	247 (95.4%)	21 (95.5%)	159 (96.4%)	67 (93.1%)	
NA	210	149	25	36	
Monocyte count, G/L	0.6 (0.5, 0.8)	0.6 (0.5, 0.8)	0.6 (0.5, 0.7)	0.8 (0.6, 0.9)	0.00079
NA	217	149	30	38	
Monocyte count, categories^a^					0.17
Monocytosis	9 (3.6%)	0 (0%)	4 (2.5%)	5 (7.1%)	
Normal	243 (96.4%)	22 (100.0%)	156 (97.5%)	65 (92.9%)	
NA	217	149	30	38	
Platelet count, G/L	294.0 (252.0, 342.0)	258.5 (243.0, 300.0)	297.0 (261.0, 352.0)	289.5 (251.0, 333.0)	0.048
NA	196	149	17	30	
CRP, mg/L	4.0 (1.0, 16.0)	1.0 (1.0, 7.0)	1.0 (1.0, 4.0)	16.0 (5.0, 35.0)	<0.0001
NA	275	157	96	22	
CRP-level, categories^a^					<0.0001
Above reference value	68 (35.1%)	3 (21.4%)	8 (8.5%)	57 (66.3%)	
Normal	126 (64.9%)	11 (78.6%)	86 (91.5%)	29 (33.7%)	
NA	275	157	96	22	
**CSF**					
CSF WBC count, cells/μL	134.5 (49.2, 254.0)		134.5 (49.2, 254.0)^b^		
NA	285	171	6	108	
CSF mononuclear fraction, %	97.7 (94.2, 99.0)		97.7 (94.2, 99.0)^b^		
NA	290	171	11	108	
CSF glucose, mmol/L	3.1 (2.8, 3.4)		3.1 (2.8, 3.4)		
NA	300	171	21	108	
Glucose index, %	57.0 (51.0, 65.2)		57.0 (51.0, 65.2)		
NA	363	171	84	108	
CSF protein, g/L	0.5 (0.3, 0.9)		0.5 (0.3, 0.9)		
NA	299	171	20	108	
CSF lactate, mmol/L	1.6 (1.3, 2.1)		1.6 (1.3, 2.1)		
NA	421	171	142	108	
CSF total IgG, g/L	0.0 (0.0, 0.1)		0.0 (0.0, 0.1)		
NA	341	171	62	108	
**Joint**					
Joint puncture	50 (10.7%)	0 (0%)	0 (0%)	50 (46.3%)^c^	
NA	361	171	190	0	
Synovial WBC count, cells/mL	40.7 (30.0, 66.9)			40.7 (30.0, 66.9)	
NA	370	171	190	9	

Continuous variables are summarized as median (1st quartile, 3rd quartile), categorical variables as no. (%). P-values were calculated by the Kruskal–Wallis rank sum test (continuous variables) or Fisher's exact test (categorical variables).

**Abbreviations:** ANC, absolute neutrophil count; CRP, C-reactive protein; CSF, cerebrospinal fluid; Ig, immunoglobulin; LNB, Lyme neuroborreliosis; NA, not available; WBC, white blood cell.

a According to age specific reference values.^31^

b A CSF pleocytosis (>5 cells/μL) was detected in 179 (97.3%) LNB patients and consisted of a lymphocytic pleocytosis (>90% mononuclear cells)^1^ in 86.4% of the cases.

c Location: knee, *n* = 43; ankle, *n* = 1; hip, *n* = 4; shoulder, *n* = 1; multiple, *n* = 1.

Among LNB patients (*n* = 190), CSF analysis results (i.e., WBC count, glucose, protein, and/or lactate levels) were available for 184 (96.8%) patients. Of the six (3.2%) patients without CSF analysis results available in medical records, five patients with meningitis underwent lumbar puncture and were diagnosed with positive two-tier serology in serum and/or CSF, and one patient with isolated FP did not have a lumbar puncture but was diagnosed with seroconversion in convalescent sera. A CSF pleocytosis (>5 cells/μL) was detected in 97.3% (*n* = 179/184) LNB patients and consisted of a lymphocytic pleocytosis (>90% mononuclear cells)^1^ in 86.4% (*n* = 159/184) of the cases. In 50 (46.3%) patients with Lyme arthritis, a joint puncture was performed due to clinical suspicion of septic arthritis (Table 4).

### Serological test results

*B. burgdorferi*-specific antibodies in initial serum samples at presentation were detected by IgM and/or IgG ELISA in 92.6% (*n* = 312/337) of patients for whom ELISA data were available (Table 5).

**Table 5:** *Borrelia burgdorferi*-specific serological testing results of 469 Swiss children and adolescents aged ≤18 years with Lyme disease grouped by skin manifestation, Lyme neuroborreliosis (LNB), and Lyme arthritis.

Characteristic	Total (*n* = 469)	Skin (*n* = 171^a^)	LNB (*n* = 190)	Arthritis (*n* = 108)	P-value
**Serum**					
Serum ELISA					<0.0001
Positive	312 (92.6%)	35 (85.4%)	169 (89.9%)	108 (100.0%)	
Negative	25 (7.4%)	6 (14.6%)	19 (10.1%)	0	
NA	132	130	2	0	
Serum ELISA categories					0.0005^b^
Positive IgM + IgG	144 (42.7%)	17 (41.5%)	95 (50.5%)	32 (29.6%)	
Positive IgM only	47 (13.9%)	1 (2.4%)	44 (23.4%)	2 (1.9%)	
Positive IgG only	108 (32.0%)	17 (41.5%)	27 (14.4%)	64 (59.3%)	
Positive (no information on isotype)	13 (3.9%)	0 (0.0%)	3 (1.6%)	10 (9.3%)	
Negative	25 (7.4%)	6 (14.6%)	19 (10.1%)	0 (0.0%)	
NA	132	130	2	0	
Serum Western blot					<0.0001
Positive	287 (88.6%)	34 (91.9%)	145 (81.0%)	108 (100.0%)	
Negative	37 (11.4%)	3 (8.1%)	34 (19.0%)	0 (0.0%)	
NA	145	134	11	0	
Serum Western blot categories					0.0005^b^
Positive IgM + IgG	126 (43.9%)	13 (38.2%)	89 (61.4%)	24 (22.2%)	
Positive IgM only	39 (13.6%)	0 (0.0%)	39 (26.9%)	0 (0.0%)	
Positive IgG only	88 (30.7%)	17 (50.0%)	8 (5.5%)	63 (58.3%)	
Positive (no information on isotype)	34 (11.8%)	4 (11.8%)	9 (6.2%)	21 (19.4%)	
Negative	37 (11.4%)	3 (8.1%)	34 (19.0%)	0 (0.0%)	
NA	145	134	11	0	
**CSF**					
CSF ELISA					
Positive	152 (86.9%)		152 (86.9%)		
Negative	23 (13.1%)		23 (13.1%)		
NA	294	171	15	108	
CSF ELISA categories					
Positive IgM + IgG	113 (64.6%)		113 (64.6%)		
Positive IgM only	18 (10.3%)		18 (10.3%)		
Positive IgG only	18 (10.3%)		18 (10.3%)		
Positive (no information on isotype)	3 (1.7%)		3 (1.7%)		
Negative	23 (13.1%)		23 (13.1%)		
NA	294	171	15	108	
Detection of intrathecal antibody production					
Positive	122 (74.8%)		122 (74.8%)		
Negative	41 (25.2%)		41 (25.2%)		
NA	306	171	27	108	

Continuous variables are summarized as median (1st quartile, 3rd quartile), categorical variables as no. (%). P-values were calculated by the Kruskal–Wallis rank sum test (continuous variables) or Fisher's exact test (categorical variables).

**Abbreviations:** CSF, cerebrospinal fluid; ELISA, enzyme-linked immunosorbent assay; Ig, immunoglobulin; LNB, Lyme neuroborreliosis; NA, not available.

a Of the patients with skin manifestations, 41 (24.0%) were serologically tested, of whom 12 had EM, 27 had lymphocytoma, and 2 had acrodermatitis chronica atrophicans. A positive serology was reported in 82.9% (*n* = 7/12) EM, 92.6% (*n* = 25/27) lymphocytoma, and 100.0% (*n* = 2/2) acrodermatitis chronica atrophicans patients. The serologies were performed on clinical suspicion, and were part of the diagnostic criteria for lymphocytoma (clinical diagnosis WITH/WITHOUT positive two-tier serology) and acrodermatitis chronica atrophicans (clinical diagnosis AND positive two-tier serology).

b P-value from Fisher's exact test calculated using Monte Carlo simulation.

Negative ELISA results in initial serum samples at presentation (*n* = 25/337, 7.4%) were observed in 6 patients with skin manifestation (*n* = 6/41, 14.6%; 4 patients with EM and 2 patients with lymphocytoma), which were clinically diagnosed according to the inclusion criteria, and in 19 patients with LNB (*n* = 19/188, 10.1%), of which 14 (*n* = 14/19, 73.7%) showed seroconversion in convalescent sera and 5 (*n* = 5/19, 26.3%) exclusively detection of antibodies in CSF.

A positive ELISA result in initial serum samples at presentation could be confirmed by Western blot in 88.6% (*n* = 287/324) patients. There were 11.4% (*n* = 37/324) patients with a negative Western blot in initial serum samples at presentation. This included 34 LNB patients (*n* = 34/37, 91.9%), of whom 15 (*n* = 15/34, 44.1%) were diagnosed serologically by IAP and/or positive ELISA and Western blot in CSF, and 19 (*n* = 19/34, 55.8%) diagnosed by seroconversion (positive ELISA and Western blot in convalescent sera). In addition, 3 patients had skin manifestations (*n* = 3/37, 8.1%) and were clinically diagnosed.

In 20 (10.5%) LNB patients, serological confirmation of the diagnosis of LNB according to the inclusion criteria was documented in the medical records at the beginning of the study period (when the microbiological reports were not yet received in electronic form), but the detailed original microbiological diagnostic reports of the *B. burgdorferi*-specific test results were not available in the medical records.

All patients with Lyme arthritis were diagnosed by positive two-tier serology in initial serum samples at presentation.

### Serological profiles of serum antibody reactivity

**Fig. 4:**
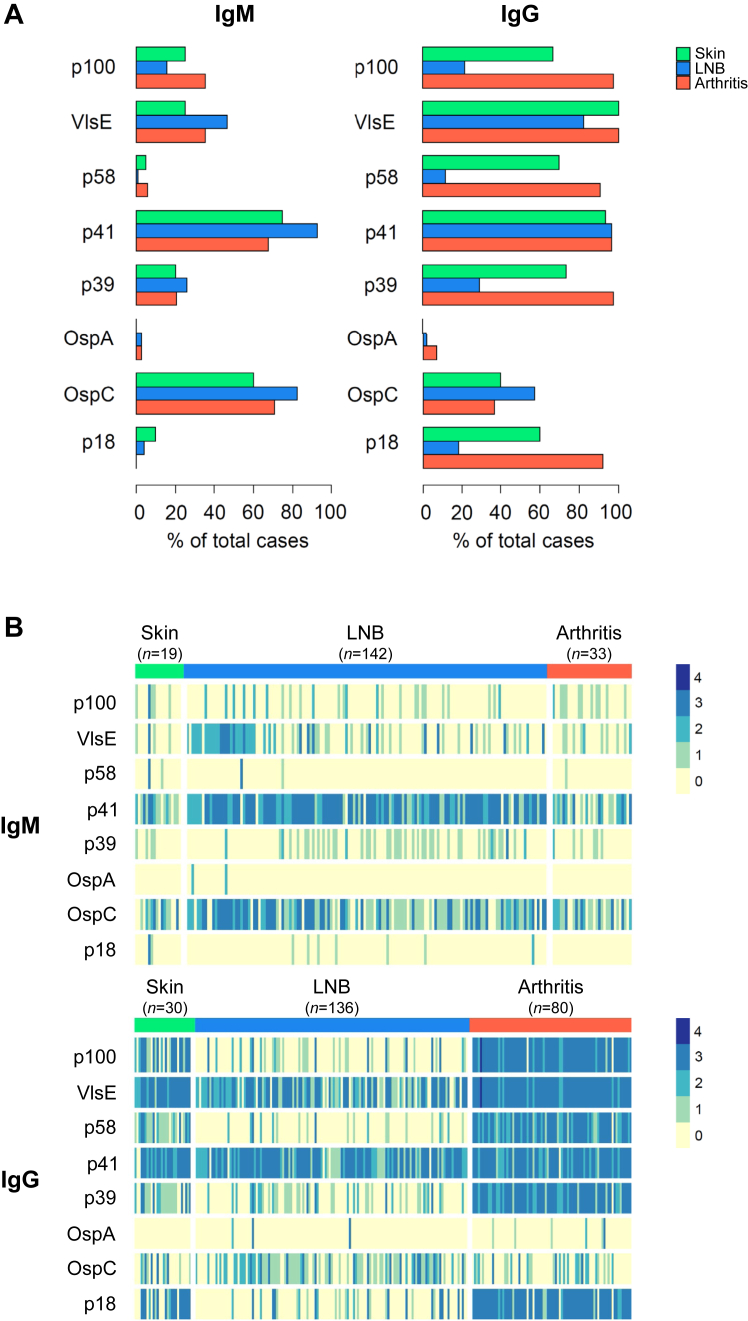
**Serum antibody responses to individual *Borrelia burgdorferi* antigens in different manifestations of Lyme disease.** Diagnostic bands for the eight antigens (p100, VlsE, p58, p41, p39, OspA, OspC, and p18) from the Western blot (*recom*Line *Borrelia* IgM and IgG; MIKROGEN Diagnostik) were assessed for color reaction and intensity after adding diluted patient serum and peroxidase conjugated anti-human antibodies (IgG or IgM). The intensity was graded as follows: 0 (no reaction, equal to the intensity of the cutoff for the control), 1+, 2+, 3+, and 4+. Intensities of at least 1+ were considered positive.^32^**A:** Percentage of positive IgM and IgG bands. **B:** Heatmap representing the intensity of IgM and IgG bands. Each column represents one patient. Western blot results for all eight antigens were available as follows: skin manifestation: IgM, *n* = 19, IgG, *n* = 30 (erythema migrans: IgM, *n* = 5, IgG, *n* = 7; lymphocytoma: IgM, *n* = 12, IgG, *n* = 21; acrodermatitis chronica atrophicans: IgM, *n* = 2, IgG, *n* = 2); Lyme neuroborreliosis (LNB): IgM, *n* = 142, IgG, *n* = 136; and Lyme arthritis: IgM, *n* = 33, IgG, *n* = 80. **Abbreviations:** Ig, immunoglobulin; LNB, Lyme neuroborreliosis.

Western blots of serum samples with results for diagnostic bands for *all* 8 *B. burgdorferi* antigens were available for 254 patients (IgM, *n* = 194; IgG, *n* = 246; Fig. 4) and used for further analyses. To compare the characteristics of this subgroup, which included results for diagnostic bands for *all* 8 *B. burgdorferi* antigens, with those of the included patients, we have listed the clinical characteristics of this subgroup in Supplementary Tables S2 and S3.

Differences were observed in specificity and concentration of *B. burgdorferi*-specific serum antibody responses among LD manifestation groups (Fig. 4). The specificity was assessed based on band positivity (band intensity of at least 1+^32^; Fig. 4A) and the concentration based on band intensity score (from 0 to 4; Fig. 4B). The band positivity varied between different manifestation groups for the isotypes (Supplementary Table S4).

Serum antibodies of the IgM isotype were mainly directed against the *B. burgdorferi* antigens p41 (range, 66.7–95.1% of total cases) and OspC (58.9–82.4%), irrespective of the affected organ system (Fig. 4A). The band intensity scores for the IgM isotype against the *B. burgdorferi* antigen VlsE, p41, and OspC were higher among LNB patients compared to the two other LD manifestation groups (Fig. 4B and Supplementary Table S4).

Serum antibodies of the IgG isotype were predominantly directed against the *B. burgdorferi* antigens p100, VlsE, p58, p41, p39, and p18 in patients with skin manifestations (majority with lymphocytoma; range, 71.4–100.0% of total cases) and Lyme arthritis (92.5–100.0%), while they were mainly directed against VlsE, p41, and OspC in LNB patients (63.2–96.3%; Fig. 4B). Patients with Lyme arthritis and skin manifestations had higher IgG band intensity scores for p100, VlsE, p58, p39, and p18 compared to LNB patients (Supplementary Table S4). Almost no reactivity was observed against OspA, p58, and p18 for IgM and OspA for IgG.

## Discussion

This is one of the largest and most detailed studies for children with LD. It shows the significant increase in the incidence of LD in children also in Switzerland. LD was mainly diagnosed from May to October, except for Lyme arthritis. Lyme arthritis was found throughout the year, which is essential for the recognition of such cases. Interestingly, patients with Lyme arthritis were older and showed higher levels of inflammatory parameters compared to children with EM or LNB. The specificity and concentration of *B. burgdorferi*-specific serum antibodies differed significantly between LD manifestations, but did not differ by age. Most importantly, the presence of pronounced serum antibody responses against the early-phase *B. burgdorferi* antigens VlsE, p41, and OspC, particularly of the IgM isotype, was associated with LNB. However, negative serological results in initial serum samples at presentation of 10.1% LNB patients demonstrated the limitations of serum serology for the diagnosis of LNB.

An increase in LD cases has also been reported for other regions in Europe and North America over the last decade, probably due to increasing spread of ticks driven by climate warming,^33^ rising prevalence of *B. burgdorferi* in ticks, or possibly also more awareness and/or better diagnostics for LD.^8^^,^^16^^,^^34^ It is important to note that our study cohort consisted of LD patients in Europe, where *Borrelia garinii* and *Borrelia afzelii* are the most common *Borrelia* genospecies and are predominantly associated with LNB and skin manifestations, respectively.^1^ *B. burgdorferi* sensu stricto is the genospecies to cause LD in the USA.^1^

The objective of this study was to better describe clinical characteristics of LD in children, as the most important step in diagnosing LD is that physicians acquire a good knowledge of the clinical features.^35^ First, we found that skin manifestations occurred also in children <5 years of age, and in half of the cases the patients recognized a tick bite around two weeks before presentation. Despite this interval, serology was negative in one third of EM cases, supporting current guidelines that recommend a purely clinical diagnosis of EM.^22^^,^^23^ Nevertheless, this clinical diagnosis remains a challenge.^36^ Serology was part of the diagnostic criteria for lymphocytoma and acrodermatitis chronica atrophicans in this study and is recommended for these manifestations according to current guidelines.^22^^,^^23^

Second, we showed that LNB occurred predominantly in 5–10-year-old children and presented by far most frequently with lymphocytic meningitis and/or FP or another cranial neuropathy, which is consistent with previous observations.^18^ Notably, the most common location of a recognized tick bite in LNB patients was most frequently the head and neck region, which supports the hypothesis that *B. burgdorferi* may also pass per continuitatem along the peripheral nerves to the central nervous system.^16^ The relatively long treatment delay of median 19 days clearly reflects the significant time required to await the two-tier serological results. Additionally, symptoms are often diffuse with an insidious onset, leading to misrecognition and delayed diagnosis due to lack of awareness about LNB manifestations among patients and/or physicians.^37^ We found persisting headache, fatigue, and fever as most common clinical symptoms of LNB. These symptoms, together with the corresponding season of the year and in regions with a high prevalence, should promptly lead to a suspected diagnosis of LNB in the presence of neurological signs suggestive of meningitis, cranial neuropathy, and/or radiculitis. Particularly the presence of FP in children should always lead to a high suspicion of LD in this situation.^18^ The frequency of LNB and presence of radiculitis is in line with the potentially greater neurotropism of *B. garinii*, which has not been isolated in North America.^1^^,^^38^ Most LNB patients showed a lymphocytic pleocytosis with >90% mononuclear cells. However, IAP was only detected in three quarters of LNB cases and exclusively seroconversion in convalescent sera was found in 7.4%, which reflects the challenges in serological diagnosis of LNB in children at presentation.

Third, patients with Lyme arthritis presented also during non-tick feeding periods in winter due to the late stage of LD, were mainly >10 years of age, and most frequently had one large joint affected, usually the knee. We found that the frequency of prolonged symptoms >2 months after completion of antibiotic treatment, referred to as antibiotic-refractory arthritis,^1^ was lower than previously reported (18% vs. 23%).^21^ Interestingly, 17% of patients diagnosed with Lyme arthritis (not all with antibiotic-refractory arthritis), which had initially fully recovered, were later diagnosed with juvenile idiopathic arthritis (data not shown), which warrants further investigation about the role of preceding *B. burgdorferi* infection and/or persisting post-infectious inflammation in these cases.^39^ Otherwise, the clinical outcome of LD in children was very good.

The shortcomings of serology in LD patients are well-known.^8^ Negative results can occur in early phases of LD, following antibiotic treatment of early *B. burgdorferi* infection, or during LNB.^37^^,^^40^, ^41^, ^42^ Positive results are observed after *B. burgdorferi* infection due to the long-lasting persistence of antibodies in serum for decades, in endemic regions with high seroprevalence (up to 50% in highly exposed populations), or with the IgM ELISA in the context of other conditions (i.e., syphilis, infectious mononucleosis, or autoimmune diseases) due to unspecific stimulation of B cells.^8^^,^^41^ The latter can be excluded by the two-tier approach.^35^ These shortcomings highlight that serology should only be performed in the presence of clinical suspicion of LD as described above (with the exception of EM).

A lumbar puncture is required for serology in CSF, which is an invasive diagnostic procedure in children, and may be questioned in view of the abovementioned limitations, especially in LNB patients with low-grade symptoms (e.g., isolated FP). Further, the newly proposed biomarker CXCL13 for LNB, which increases in CSF even before IAP detection and rapidly decreases during antibiotic treatment, is also not increased in LNB without IAP detection and can also be detected in other disorders (e.g., neurosyphilis and CNS lymphoma).^43^

To date, there is no single set of criteria to interpret Western blot results with high levels of sensitivity and specificity.^8^ However, we observed a distinct IgM Western blot pattern for LNB including pronounced serum antibody responses against the early-phase *B. burgdorferi* surface antigens VlsE (variable major protein-like sequence expressed), p41 (flagellin protein), and OspC (outer surface protein C). VlsE represents immunodominant epitopes of different genospecies implicated in infection and immune evasion, and OspC plays an important role during infection.^44^ VlsE and OspC are considered (highly) specific, while p41 has been found to cross-react with other flagellated bacteria and detected in half of healthy individuals.^32^ A previous study corroborated our findings by showing that a panel containing these three antigens may improve diagnosis for early disseminated LD.^44^ In line with our results, this study also found differential serologic correlates of LD stage, suggesting a role for the host immune response in clinical presentation.

Considering the increase in prevalence of LD and the challenges in diagnosing LD, there is a need for improved diagnostic methods that are capable of reliably detecting *B. burgdorferi* infection at all disease stages. A multifaceted effort is needed to advance research about ticks, *B. burgdorferi* genospecies, and most importantly, the pathophysiology of LD in children.^34^ In fact, excessive immune responses, rather than direct damage caused by *B. burgdorferi*, is assumed to be the main pathological mechanism contributing to LD, particularly in LNB and Lyme arthritis.^12^^,^^18^^,^^37^^,^^39^ This may be in line with the laboratory and immunological findings of this study. In particular, B cells seem to play a crucial role in the resolution of symptoms and disease, as well as in bacterial clearance.^45^ A previous study identified plasmablasts as an essential B-cell population in LD which correlated with rapid resolution of symptoms in adults.^46^ Pathogen-specific plasmablasts can be easily detected in a clinical setting during various infectious diseases^47^^,^^48^ and its measurement has been shown to improve diagnosis in childhood pneumonia caused by *Mycoplasma pneumoniae*.^48^ Thus, its detection may be also a resource for determining disease aetiology in LD, particularly in early (disseminated) stages such as LNB. Another new diagnostic approach is the use of proteomics with high-resolution tandem mass spectrometry to detect *Borrelia* peptides, which is showing promising preliminary results.^49^

The strengths of this study are the large number of included children with LD, the clearly defined study population, and the use of the Western blot *recom*Line *Borrelia* IgM and IgG^32^ over the whole 15-year study period. The study has several limitations. First, as with studies of LD in general, a major problem is the underestimation of cases, which are also spread across age groups and countries. This problem greatly reduces the statistical value of any findings. Second, the study design is retrospective. Thus, we were not able to provide total numbers about negative or not tested patients. Moreover, not all data were available for all patients and some patients were lost to follow-up. We had standardized follow-up visits over the 15-year study period at the end of treatment for patients with LNB other than isolated FP, and at 10 days and 4 weeks after the onset of FP for patients with isolated FP. Otherwise, follow-up visits were individually planned and conducted differently in accordance with clinical manifestations. It is therefore possible that the retrospective study design, without standardized follow-up visits also at later time points, may have failed to detect or record minor deficits that are rarely reported in LNB and do not appear to interfere with normal activities.^14^ Non-specific, subjective symptoms (e.g., fatigue, headache, problems with concentration or memory) were previously reported in children diagnosed with LNB after treatment as often as in healthy age-matched controls.^50^ Third, the patient cohort is geographically confined to the region of Zurich, Switzerland. Finally, the number of patients with less-severe LD manifestations such as EM or lymphocytoma is probably underestimated, as these patients are mainly managed by general practitioners. In addition, the study is monocentric and the hospital is an important pediatric reference center, so the cases analyzed tend to have even a higher clinical severity.

In conclusion, this study of LD in children showed that clinical characteristics and specific serum antibody response patterns differed between LD manifestations. We found that distinct patterns of pronounced serum antibody responses of the IgM isotype against three *B. burgdorferi* antigens (VlsE, p41, and OspC) may help to predict LNB, and of the IgG isotype against six *B. burgdorferi* antigens (p100, VlsE, p58, p41, p39, and p18) were associated with Lyme arthritis. Considering the diagnostic challenges in LD, and especially LNB, there is a need to advance current testing methods while exploring new and innovative diagnostic approaches.

## Contributors

**Study concept and design**: P.M.M.S., C.B., and approved by all authors; **Acquisition of data**: B.M.G., M.M., A.E., and P.M.M.S.; **Analysis and interpretation of data**: B.M.G., P.M.M.S., S.S., E.O., S.v.F., and C.B.; **Drafting of the manuscript**: B.M.G. and P.M.M.S.; **Critical revision of the manuscript for important intellectual content**: all authors; **Statistical analysis**: B.M.G. and S.v.F.; **Obtained funding**: P.M.M.S. and S.S.; **Administrative, technical, or material support**: P.M.M.S., S.S., M.M., A.E., and C.B.

## Data sharing statement

B.M.G. and P.M.M.S. have full access to all of the data in the study and take responsibility for the integrity of the data and the accuracy of the data analysis. Data reported in this study are available within the article and/or the Supplementary Material. More information is available from the corresponding author on reasonable request from any qualified investigator.

## Declaration of interests

In the past 36 months, P.M.M.S. has served on advisory boards (Roche, Sanofi, AstraZeneca) and given presentations (Fomf, FPH Forum, ZAIM MediDays, Insight Paediatrics) with payments to the institution (University Children's Hospital Zurich). A.E. has received consultancy fees from Sefunda and Phast (advisory role to start-up companies), honoraria for presentations at various conferences at industry sponsored symposia (lllumina, Copan, Bruker), and support from the Laboratory Medicine Society of Korea for attendance at and travel to the annual meeting of the Laboratory Medicine Society of Korea in the past 36 months. All authors have submitted the ICMJE Form for Disclosure of Potential Conflicts of Interest.
