# Supplementary material for: Clinical characteristics and serological profiles of Lyme disease in children: a 15-year retrospective cohort study in Switzerland
Source: Lancet Reg Health Eur. 2024 Dec 9;48:101143. doi: 10.1016/j.lanepe.2024.101143 (PMC11683244; doi:10.1016/j.lanepe.2024.101143)

Supplementary data

Table of contents

**Table S1:**	Clinical characteristics of peripheral facial nerve palsy at presentation of the whole cohort	Page 2
**Table S2**	Demographic characteristics and clinical presentation of the subcohort of 254 patients with Lyme disease and complete Western blot results for all 8 *B. burgdorferi* antigens	Page 3
**Table S3**	Treatment and clinical outcome of the subcohort of 254 patients with Lyme disease and complete Western blot results for all 8 *B. burgdorferi* antigens	Page 6
**Table S4**	Western blot band intensity score (0–4) of the subcohort of 254 patients with Lyme disease and complete Western blot results for all 8 *B. burgdorferi* antigens	Page 8
**Figure S1**	Presence of constitutional symptoms of the whole cohort of 469 patients with different manifestations of Lyme disease	Page 10
**Figure S2**	White blood cell count and C-reactive protein levels of the whole cohort of 469 patients with different manifestations of Lyme disease	Page 11

Supplementary Table S1. Clinical characteristics of peripheral facial nerve palsy at presentation of the whole cohort.

**Clinical characteristics of peripheral facial nerve palsy (FP)**	***n*[1]**
**Total**	
	136[a]
**Side**	
Right	70 (51.0%)
Left	62 (46.0%)
Bilateral	3 (2.2%)
NA	1 (0.7%)
**Grading with House-Brackmann classification**	
II mild dysfunction	12 (8.8%)
III moderate dysfunction	31 (23.0%)
IV moderately severe dysfunction, incomplete eye closure	71 (52.0%)
V severe dysfunction	17 (13.0%)
NA	5 (3.7%)

Categorical variables are summarized as no. (%).

Abbreviations: FP, peripheral facial nerve palsy; NA, not available.

[a] Including isolated FP (*n*=63; defined as FP without any other medical symptoms and signs such as headache, fever, nuchal rigidity etc.), FP with meningitis (*n*=69), FP with (meningo-)radiculitis (*n*=3), and FP with cerebral vasculitis (*n*=1) (**Table 1**).

Supplementary Table S2. Demographic characteristics and clinical presentation of the subcohort of 254 patients with Lyme disease and complete Western blot results for all 8 *B. burgdorferi* antigens.

Characteristics	Total (*n*=254)	Skin (*n*=30)	LNB (*n*=143)	Arthritis (*n*=81)
**Demographic characteristics**				
Age (years)	8.3 (6.0,10.8)	7.1 (4.5,10.5)	7.6 (5.8,9.8)	10.1 (8.3,12.3)
Sex, male	154 (60.6%)	20 (66.7%)	86 (60.1%)	48 (59.3%)
Underlying disease	33 (13.0%)	6 (20.7%)	16 (11.2%)	11 (13.6%)
Underlying disease				
Primary or secondary immunodeficiency	0 (0%)	0 (0%)	0 (0%)	0 (0%)
Neurological	9 (27.3%)	1 (16.7%)	6 (37.5%)	2 (18.2%)
Cardiovascular	2 (6.1%)	0 (0.0%)	1 (6.3%)	1 (9.1%)
Pulmonary	5 (15.2%)	2 (33.3%)	2 (12.5%)	1 (9.1%)
Gastrointestinal	2 (6.1%)	0 (0.0%)	1 (6.3%)	1 (9.1%)
Others	15 (45.5%)	3 (50.0%)	6 (37.5%)	6 (54.5%)
No underlying disease	221	24	127	70
Exposition to tick bite				
Farm	3 (5.8%)	2 (15.4%)	0 (0.0%)	1 (16.7%)
Forest	29 (55.8%)	5 (38.5%)	21 (63.6%)	3 (50.0%)
Garden	3 (5.8%)	0 (0.0%)	3 (9.1%)	0 (0.0%)
Hiking	2 (3.8%)	0 (0.0%)	2 (6.1%)	0 (0.0%)
Scout	0 (0.0%)	0 (0.0%)	0 (0.0%)	0 (0.0%)
Unknown exposition	15 (28.8%)	6 (46.2%)	7 (21.2%)	2 (33.3%)
NA	202	17	110	75
Tick bite recognized	103 (40.6%)	15 (51.7%)	65 (45.5%)	23 (28.8%)
Location of recognized tick bite				
Head/neck	22 (21.4%)	6 (40.0%)	16 (24.6%)	0 (0.0%)
Trunk	11 (10.7%)	0 (0.0%)	11 (16.9%)	0 (0.0%)
Upper limb	2 (1.9%)	1 (6.7%)	1 (1.5%)	0 (0.0%)
Lower limb	1 (1.0%)	0 (0.0%)	1 (1.5%)	0 (0.0%)
Multiple tick bites	6 (5.8%)	2 (13.3%)	2 (3.1%)	2 (8.7%)
Unknown localization	61 (59.2%)	6 (40.0%)	34 (52.3%)	21 (91.3%)

**Characteristics**	**Total** (*n*=254)	**Skin** (*n*=30)	**LNB** (*n*=143)	**Arthritis** (*n*=81)
NA	151	15	78	58
EM recognized	29 (11.4%)	6 (20.0%)	22 (15.4%)	1 (1.2%)
Location of recognized EM				
Head/neck	15 (51.7%)	3 (50.0%)	12 (54.5%)	0 (0.0%)
Trunk	7 (24.1%)	2 (33.3%)	5 (22.7%)	0 (0.0%)
Upper limb	0 (0.0%)	0 (0.0%)	0 (0.0%)	0 (0.0%)
Lower limb	0 (0.0%)	0 (0.0%)	0 (0.0%)	0 (0.0%)
Multiple EM	4 (13.8%)	1 (16.7%)	3 (13.6%)	0 (0.0%)
Unknown localization	3 (10.3%)	0 (0.0%)	2 (9.1%)	1 (100.0%)
NA	225	24	121	80
**Clinical presentation**				
Time from recognized tick bite to ED presentation (days)	43.0 (30.0,300.0)	60.0 (43.0,120.0)	30.0 (27.0,60.0)	360.0 (140.0,720.0)
NA	167	19	84	64
Time from symptom onset to ED presentation (days)	7.0 (3.0,21.0)	71.5 (34.0,140.0)	7.0 (3.0,14.0)	3.0 (2.0,10.0)
Fever	43 (16.9%)	3 (10.0%)	28 (19.6%)	12 (14.8%)
Headache	90 (35.4%)	1 (3.3%)	86 (60.1%)	3 (3.7%)
Nausea/vomit	21 (8.3%)	0 (0.0%)	21 (14.7%)	0 (0.0%)
Nuchal rigidity	26 (10.3%)	0 (0.0%)	26 (18.4%)	0 (0.0%)
Scalp touch sensitive	9 (3.6%)	0 (0.0%)	9 (6.3%)	0 (0.0%)
Fatigue	50 (19.8%)	1 (3.3%)	49 (34.5%)	0 (0.0%)
Change in behavior/character	24 (9.4%)	1 (3.3%)	23 (16.1%)	0 (0.0%)
EM at ED presentation	24 (9.4%)	5 (16.7%)	19 (13.3%)	0 (0.0%)
Location of EM at ED presentation				
Head/neck	16 (66.7%)	3 (60.0%)[a]	13 (68.4%)[a]	
Trunk	2 (8.3%)	0 (0.0%)	2 (10.5%)	
Upper limb	2 (8.3%)	1 (20.0%)	1 (5.3%)	
Lower limb	0 (0.0%)	0 (0.0%)	0 (0.0%)	
Multiple EM	4 (16.7%)	1 (20.0%)	3 (15.8%)	
Unknown localization	0 (0.0%)	0 (0.0%)	0 (0.0%)	
NA	230	25	124	81

Characteristics	Total (*n*=254)	Skin (*n*=30)	LNB (*n*=143)	Arthritis (*n*=81)
Lymphocytoma at presentation	21 (8.3%)	20 (66.7%)	1 (0.7%)	0 (0.0%)
Location of Lymphocytoma at ED presentation				
Ear lobe	14 (66.7%)	13 (65.0%)	1 (100.0%)	
Supraorbital	2 (9.5%)	2 (10.0%)	0 (0.0%)	
Mamilla	3 (14.3%)	3 (15.0%)	0 (0.0%)	
Cheek	2 (9.5%)	2 (10.0%)	0 (0.0%)	
Unknown localization	0 (0.0%)	0 (0.0%)	0 (0.0%)	
NA	233	10	142	81

Continuous variables are summarized as median (1st quartile, 3rd quartile), categorical variables as no.

Abbreviations: ED, emergency department; EM, erythema migrans; LNB, Lyme neuroborreliosis; NA, not available.

[a] Including erythema on the cheeks (which resembles slapped-cheek rash from fifth disease due to parvovirus B19 infection).

Supplementary Table S3. Treatment and clinical outcome of the subcohort of 254 patients with Lyme disease and complete Western blot results for all 8 *B. burgdorferi* antigens.

**Characteristic**	**Total** (*n*=254)	**Skin** (*n*=30)	**LNB** (*n*=143)	**Arthritis** (*n*=81)
**Treatment**				
Prior antibiotic treatment for LD	15 (5.9%)	2 (6.7%)	10 (7.0%)	3 (3.7%)
Prior antibiotic treatment duration (days)	4.0 (3.0,7.0)	9.5 (7.0,12.0)	4.0 (3.0,5.0)	12.0 (4.0,20.0)
NA	241	28	134	79
Time from symptom onset to start of antibiotic treatment after presentation (days)	19.0 (12.0,35.0)	78.5 (34.0,142.0)	19.0 (13.0,30.0)	12.5 (9.0,25.0)
NA	13	2	10	1
Antibiotic treatment after presentation				
Amoxicillin PO	34 (13.9%)	22 (75.9%)	0 (0.0%)	12 (14.8%)
Ceftriaxone IV[a]	110 (45.1%)	0 (0.0%)	106 (79.1%)	4 (4.9%)
Doxycycline PO	100 (41.0%)	7 (24.1%)	28 (20.9%)	65 (80.2%)
Other antibiotic treatment	0 (0.0%)	0 (0.0%)	0 (0.0%)	0 (0.0%)
NA	10	1	9	0
Antibiotic treatment duration (days)	21.0 (14.0,28.0)	21.0 (14.0,21.0)	21.0 (14.0,21.0)	28.0 (28.0,28.0)
NA	11	1	10	0
Side effects[b]				
Amoxicillin	0 (0.0%)		0 (0.0%)	
Ceftriaxone	15 (6.1%)		15 (11.2%)	
Amoxicillin with clavulanic acid	0 (0.0%)		0 (0.0%)	
Doxycycline	0 (0%)		0 (0%)	
No side effects	229 (93.9%)	30 (100.0%)	119 (88.8%)	81 (100.0%)
NA	10	1	9	0
Change of antibiotics				
to Doxycycline	3 (75.0%)		3 (75.0%)	
to Penicillin	1 (25.0%)		1 (25.0%)	
NA	250	30	139	81
Prior corticosteroid treatment	8 (3.1%)	0 (0.0%)	8 (5.6%)	0 (0%)

Characteristic	Total (*n*=254)	Skin (*n*=30)	LNB (*n*=143)	Arthritis (*n*=81)
Corticosteroids at presentation	86 (33.9%)		86 (62.3%)[c]	
**Clinical outcome**				
Hospitalization	31 (12.2%)	0 (0.0%)	7 (4.9%)	24 (29.6%)
Duration of hospital stay (days)	5.0 (3.0,7.0)		6.0 (3.0,10.0)	5.0 (3.0,6.0)
NA	223	30	136	57
Total duration of symptoms (days)	40.5 (24.0,64.0)	50.5 (34.0,132.5)[d]	33.0 (23.0,54.0)	45.0 (30.0,108.0)
NA	84	18	56	10
Presence of prolonged symptoms				
2 to 3 months	1 (5.3%)	0 (0.0%)		1 (6.7%)
4 to 6 months	8 (42.1%)	3 (75.0%)		5 (33.3%)
>6 months	10 (52.6%)	1 (25.0%)		9 (60.0%)
No prolonged symptoms	198 (91.2%)	21 (84.0%)	114 (100.0%)	63 (80.8%)
NA	235	26	143	66
Clinical outcome at last follow-up visit				
Full recovery	217 (99.5%)	25 (100.0%)	114 (99.1%)	78 (100.0%)
Abnormal clinical outcome[e]	1 (0.5%)	0 (0.0%)	1 (0.9%)	0 (0.0%)
NA	36	5	28	3

Continuous variables are summarized as median (1st quartile, 3rd quartile), categorical variables as no.

Abbreviations: LD, lyme disease; IV, intravenous; LNB, Lyme neuroborreliosis; NA, not available; PO, peroral.

[a] Ceftriaxone IV once daily was administered on an outpatient basis at our day clinic for the entire duration of the study, unless the patient's general condition was severely compromised and required hospitalisation with monitoring.

[b] Ceftriaxone: exanthema (*n*=8), angioedema (*n*=1), fever (*n*=2), neutropenia (*n*=3), elevated transaminases (*n*=1).

[c] According to treatment recommendations indicated for and administered to the following patients: cranial neuropathies (if ≤5 days of symptoms), *n*=84; meningoradiculitis, *n*=1; and myelitis, *n*=1 (table 1).

[d] EM (*n*=3), 34 (28–38); lymphocytoma (*n*=8), 55 (43–128); acrodermatitis chronica atrophicans (*n*=1), 437 (437–437).

[e] Abnormal clinical outcome: cerebral vasculitis with cerebrovascular insult (*n*=1).

Supplementary Table S4. Western blot band intensity score (0–4) of the subcohort of 254 patients with Lyme disease and complete Western blot results for all 8 *B. burgdorferi* antigens.

**Characteristic**	**Total** (*n*=254)	**Skin** (*n*=30)	**LNB** (*n*=143)	**Arthritis** (*n*=81)	**p-value**
**IgM**					
IgM p100	0 (0,0)	0 (0,1)	0 (0,0)	0 (0,1)	0.083
NA	60	11	1	48	
IgM VlsE	0 (0,1)	0 (0,0)	0 (0,2)	0 (0,1)	0.038
NA	60	11	1	48	
IgM p58	0 (0,0)	0 (0,0)	0 (0,0)	0 (0,0)	0.055
NA	60	11	1	48	
IgM p41	2 (1,3)	1 (0,2)	3 (2,3)	1 (0,2)	<0.0001
NA	60	11	1	48	
IgM p39	0 (0,1)	0 (0,0)	0 (0,1)	0 (0,0)	0.43
NA	60	11	1	48	
IgM OspA	0 (0,0)	0 (0,0)	0 (0,0)	0 (0,0)	0.67
NA	60	11	1	48	
IgM OspC	1 (1,3)	1 (0,2)	2 (1,3)	1 (0,2)	0.015
NA	60	11	1	48	
IgM p18	0 (0,0)	0 (0,0)	0 (0,0)	0 (0,0)	0.20
NA	60	11	1	48	
**IgG**					
IgG p100	1 (0,3)	2 (0,3)	0 (0,0)	3 (3,3)	<0.0001
NA	6	0	6	0	
IgG VlsE	3 (2,3)	3 (3,3)	2 (1,3)	3 (3,3)	<0.0001
NA	7	0	7	0	
IgG p58	0 (0,2)	1 (1,2)	0 (0,0)	3 (2,3)	<0.0001
NA	7	0	6	1	
IgG p41	3 (2,3)	3 (2,3)	3 (2,3)	3 (2,3)	0.063
NA	7	0	6	1	

**Characteristic**	**Total** (*n*=254)	**Skin** (*n*=30)	**LNB** (*n*=143)	**Arthritis** (*n*=81)	**p-value**
IgG p39	1 (0,3)	1 (0,2)	0 (0,1)	3 (2,3)	<0.0001
NA	6	0	6	0	
IgG OspC	1 (0,2)	0 (0,1)	1 (0,2)	0 (0,1)	0.0028
NA	6	0	6	0	
IgG OspA	0 (0,0)	0 (0,0)	0 (0,0)	0 (0,0)	0.079
NA	6	0	6	0	
IgG p18	1 (0,3)	3 (0,3)	0 (0,0)	3 (3,3)	<0.0001
NA	6	0	6	0	

Continuous variables are summarized as median (1st quartile, 3rd quartile). P-values were calculated by the Kruskal-Wallis rank sum test (continuous variables).

Abbreviations: Ig, immunoglobulin; LNB, Lyme neuroborreliosis; NA, not available.

Supplementary Figure S1. Presence of constitutional symptoms of the whole cohort of 469 patients with different manifestations of Lyme disease.

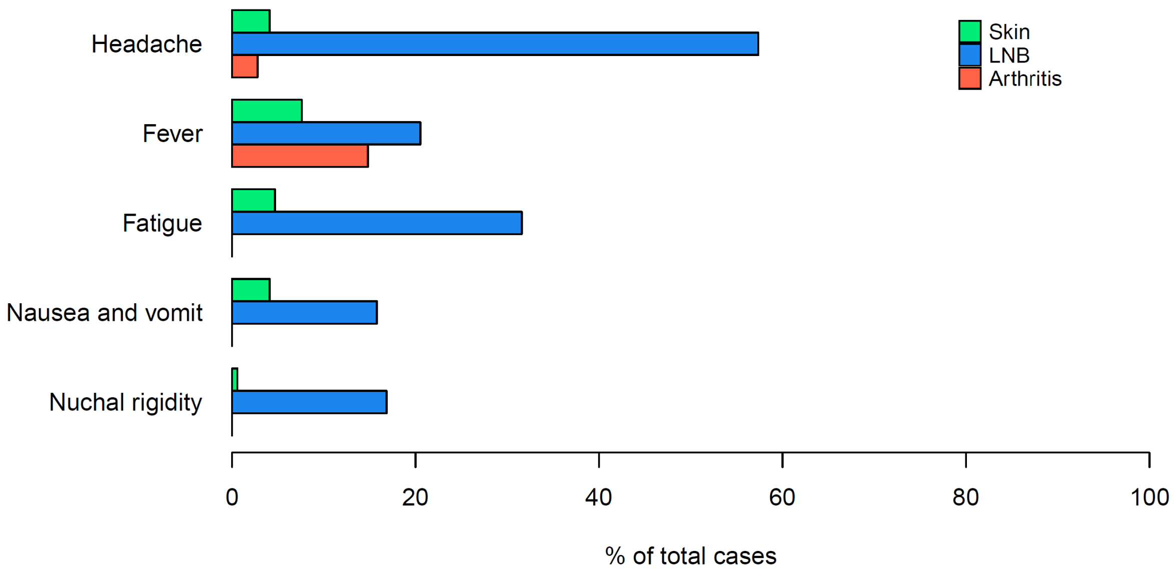

Supplementary Figure S2. White blood cell count and C-reactive protein levels of the whole cohort of 469 patients with different manifestations of Lyme disease. ***A:*** WBC count in blood. ***B:*** C-reactive protein (CRP) levels in blood. The median is shown as a black line across the box that represents the lower and upper quartiles. Whiskers extend to the maximum and minimum values within 1.5 times the interquartile range above and below the third and first quartiles, respectively. **Abbreviations:** CRP, C-reactive protein; LNB, Lyme neuroborreliosis, WBC, white blood cell.

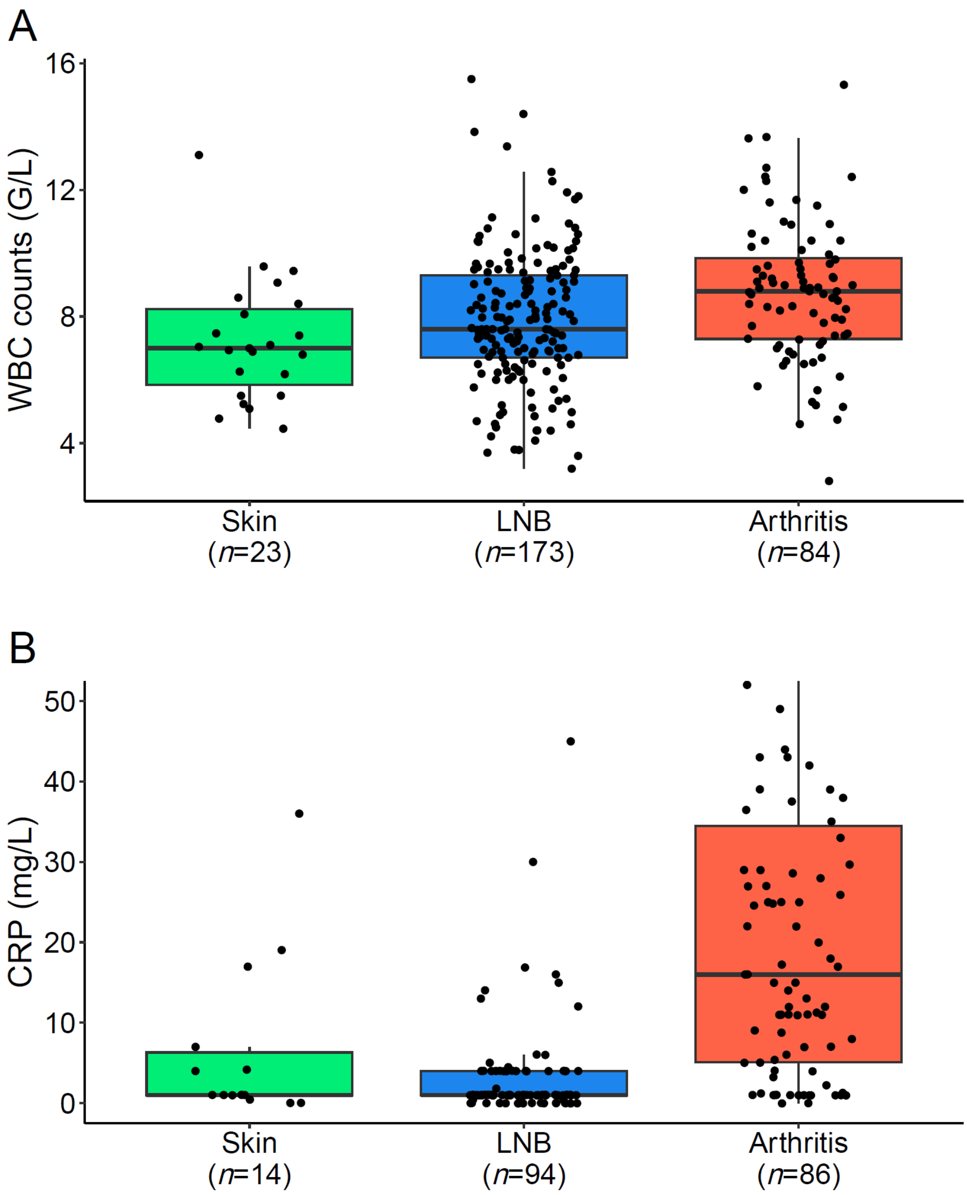

Supplement: Supplementary Tables S1–S4 and Figures S1 and S2 [file mmc1.pdf]
